# How We Treat Hemolytic Anemia Due to Pyruvate Kinase Deficiency

**DOI:** 10.3390/hematolrep16030054

**Published:** 2024-08-31

**Authors:** Sara Tama-Shekan, Valeria Moreno, Ludovic Saba, Chakra P. Chaulagain

**Affiliations:** Department of Hematology and Oncology, Maroone Cancer Center, Cleveland Clinic Florida, Weston, FL 33331, USA; s.tamashekan@mua.edu (S.T.-S.); morenov@ccf.org (V.M.); sabal2@ccf.org (L.S.)

**Keywords:** pyruvate kinase deficiency, hemolytic anemia, mitapivat, transfusional iron overload

## Abstract

Background: Pyruvate kinase (PK) deficiency is an inherited red blood cell (RBC) enzyme disorder that results in non-immune chronic hemolytic anemia. Characteristic symptoms of PK deficiency include anemia, fatigue, splenomegaly, jaundice, gallstones, thrombosis, and transfusional iron overload. Previously, treatments aimed at symptomatic management with RBC transfusions, phototherapy, folic acid supplementation, splenectomy, and iron chelation therapy when iron overload was documented. Mitapivat, a recently approved medication for treatment of PK-deficiency hemolytic anemia, is an oral allosteric activator of wild-type and mutant RBC PK enzymes. In this paper, we describe three cases of PK-deficiency anemia treated with mitapivat and describe modern management of this rare hemolytic disorder. Methods: A retrospective healthcare database analysis was conducted to extract relevant information. Both quantitative and qualitative methods were integrated to provide a more comprehensive understanding of the cases. Results: Two patients responded well to treatment with mitapivat, noted by an increase in hemoglobin levels, improvements in hemolytic markers, less frequent or no RBC transfusion requirements, and improvements in fatigue. One patient carrying two non-missense mutations of the *PKLR* gene did not respond to treatment with mitapivat. As variations in patient-specific factors (including genotype) can lead to different clinical manifestations and responses to treatment, we recommend considering both the phenotype (clinical symptoms and signs) and the genotype of the *PKLR* gene when making therapeutic decisions about starting a patient on mitapivat. Conclusions: While mitapivat addresses the previously unmet needs of most patients with PK deficiency as the first and only disease-modifying medication to receive approval for this condition, not all patients with PK deficiency are amenable to treatment with mitapivat.

## 1. Introduction

Pyruvate kinase (PK) deficiency is an inherited red blood cell (RBC) enzyme disorder that results in non-immune chronic hemolytic anemia. It is a lifelong illness with several complications. PK is a crucial enzyme in the glycolytic pathway that is responsible for efficient generation of adenosine triphosphate (ATP); its absence or reduced activity leads to the premature breakdown of RBCs. PK deficiency has an autosomal recessive inheritance pattern, caused by one or more mutations of the *PKLR* gene, resulting in characteristic symptoms such as anemia, fatigue, splenomegaly, jaundice, gallstones, thrombosis, and transfusional iron overload [1]. The notable clinical heterogeneity of this disorder is likely attributable to the vast variety of *PKLR* gene mutations that impact PK function differently in different individuals [2]. Critical complications in individuals with PK deficiency include severe hemolysis necessitating splenectomy, gallstones requiring cholecystectomy, thrombosis, liver cirrhosis resulting from iron overload, and extramedullary hematopoiesis. Prior to the approval of mitapivat, an oral allosteric activator of RBC PK, by the Food and Drug Administration (FDA) in February 2022, there was no specific treatment for hemolytic anemia in adults with PK deficiency, and treatment options were aimed at symptomatic and supportive care [3]. In a phase 3 randomized placebo-controlled trial involving patients with PK deficiency, mitapivat significantly increased hemoglobin levels and demonstrated early and meaningful improvement of key markers of hemolysis, such as indirect bilirubin, lactate dehydrogenase (LDH), and haptoglobin levels, as well as hematopoietic activity (reticulocyte %) [4]. The most favorable responses to mitapivat are observed in individuals carrying missense mutations in the *PKLR* gene, while individuals who are homozygous for *R479H* or possess two non-missense mutations have lower response rates or do not respond to treatment at all [5]. We report two cases of patients with PK deficiency who possess missense/non-missense mutations and one case of a patient with non-missense/non-missense *PKLR* gene mutation with varying presentations of hemolytic anemia and treated with mitapivat and discuss their responses to therapy. The patient characteristics are presented in Table 1.

## 2. Case Report

### 2.1. Case 1

In September 2016, an 18-year-old white male sought care for PK deficiency leading to hemolytic anemia. At birth, he presented with prolonged neonatal hemolytic jaundice, needing several units of RBC transfusions, until 14 months of age. He had a history of multiple gallstones and underwent cholecystectomy at the age of 11. At presentation, the patient reported normal energy levels. On physical examination, scleral icterus was noted. Hemoglobin was 9.5 g/dL (range: 13.2–17.1 g/dL), LDH was 648 U/L (range: 135–225 U/L), and total bilirubin was 10.0 mg/dL (range: 0.2–1.2 mg/dL). Iron saturation and ferritin were within normal limits. The patient was taking folic acid 1mg daily. PK diagnosis was confirmed with low PK levels and genetic testing that demonstrated heterozygous *PKLR* gene mutations: a c.721G>T nonsense mutation on exon 6 and a c.1484C>T missense mutation on exon 10.

In February 2021, hemoglobin had dropped to 6.9 g/dL and total bilirubin was 7.5 mg/dL; the patient subsequently received two units of RBC transfusions. The following year, in February 2022, the patient’s hemoglobin dropped again to 6.8 g/dL, total bilirubin was 6.7 mg/dL, and the patient received one unit of RBC transfusion. In March 2022, PK enzyme activity was 1.8 U/g Hb (range: 5.5–12.4 U/g Hb). By July 2022, hemoglobin had dropped to 6.8 g/dL again, causing symptomatic anemia, and the patient received two units of RBC transfusions.

**Figure 1:**
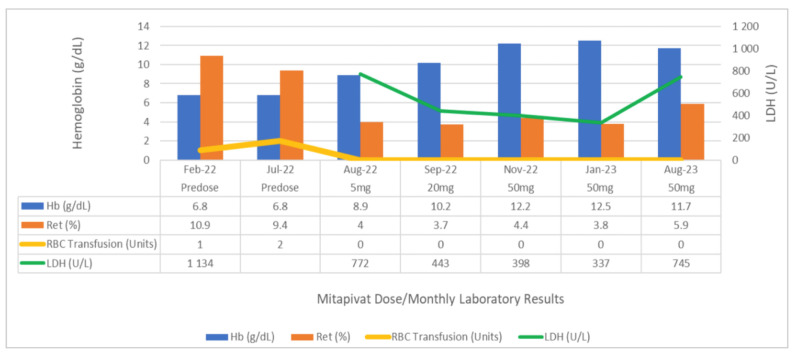
Comparison of hemoglobin, reticulocytes, LDH levels, and RBC transfusion requirements, pre-dose and between mitapivat doses in case 1. LDH = lactate dehydrogenase; Hb = hemoglobin; RBC = red blood cell; Ret = reticulocytes.

At this point, the patient had become transfusion-dependent, there was notable moderate splenomegaly on examination, and the decision was made to initiate therapy with mitapivat. The goal of therapy was to obviate the need for splenectomy and to prevent transfusional iron overload in this patient, who had not yet developed iron overload from transfusion but was at risk. In July 2022, mitapivat was initiated at 5mg twice daily for one month. In August 2022, repeat hemoglobin was 8.9 g/dL, and mitapivat was increased to 20mg twice daily, according to the recommended dose escalation. In September 2022, hemoglobin improved to 10.4 g/dL, and therapy was increased to 50 mg of mitapivat, twice daily, the maximum recommended dose. Since initiation of treatment, hemoglobin has steadily increased, with the most current hemoglobin being 11.7 g/dL, total bilirubin being 4.8 mg/dL, LDH being 745 U/L, and reticulocytes being 5.9% (Figure 1). The patient has not required any RBC transfusions since initiation of therapy, has improved fatigue, and is able to work full-time. He continues to tolerate therapy and reports compliance.

### 2.2. Case 2

In September 2019, a 52-year-old Hispanic male sought care for anemia and iron overload. The patient was diagnosed with PK deficiency at birth following episodes of hemolysis- and transfusion-dependency until he underwent splenectomy at the age of 2. Following multiple biliary pigment stones, he underwent cholecystectomy at the age of 15. At the age of 18, the patient had a mesenteric artery thrombosis that required a small bowel resection. The patient required intermittent blood transfusions into adult life that resulted in secondary iron overload and cirrhosis of the liver, confirmed by a liver biopsy in 2002. Subsequently, the patient was treated with frequent phlebotomies along with iron chelation therapy with an intraperitoneal pump. The phlebotomies were discontinued due to severe anemia a few years after initiation. Between 2012 and 2018, the patient had multiple episodes of severe cholangitis complicated by sepsis requiring repeated endoscopic retrograde cholangiopancreatography (ERCP) for bile duct stone removals, complicated by secondary sclerosing cholangitis. In June 2019, the patient had choledocholithiasis and underwent balloon extraction.

When the patient presented to our clinic in 2019, he reported intermittent palpitations and mild chronic fatigue. The physical exam was significant for scleral icterus and appreciable firm hepatomegaly. Hemoglobin was 9.1 g/dL (range: 13.2–17.1 g/dL), platelets were 674 (range: 140–400 Thousand/μL), LDH was 229 U/L (range: 135–225 U/L), total bilirubin was 5.7 mg/dL (range: 0.2–1.2 mg/dL), and direct bilirubin was 0.9 mg/dL (range: <0.2 mg/dL). The patient was taking folic acid 1mg daily. In January 2020, further evaluation revealed an elevated ferritin level of 2374 ng/mL (range: 30.3–565.7 ng/mL) and an iron saturation of 92%. The decision was made to re-initiate iron chelation therapy with deferasirox at 50% dose reduction to adjust for hepatotoxic potential in the setting of cirrhosis of the liver due to transfusional iron overload. From September 2020 to September 2021, the patient was admitted to the hospital for two recurrent episodes of cholangitis.

By September 2022, hemoglobin was 9.0 g/dL, total bilirubin was 5.2 mg/dL, and the patient remained iron-overloaded despite iron chelation therapy. He was also experiencing significant fatigue secondary to anemia and worsening underlying chronic liver failure. Enzyme and genetic testing confirmed the diagnosis of PK deficiency with heterozygous *PKLR* gene mutation—a c.1091dup frameshift mutation on exon 7 and a c.1493G>A missense mutation on exon 10. In November 2022, while the patient was waiting for mitapivat to arrive, he underwent an orthotopic liver transplant for acute on-chronic liver failure secondary to parenchymal liver disease from iron overload and biliary cirrhosis from chronic recurrent choledocholithiasis and bacterial cholangitis. This hospitalization was complicated by acute kidney injury (hepatorenal syndrome), which improved after the liver transplantation. Over the course of one month after liver transplantation, the patient required a total of 10 units of RBC transfusions, and mitapivat therapy was initiated at 5 mg, twice daily. The goal of therapy was to improve hemoglobin and decrease the transfusion burden. Furthermore, improvement in iron overload from chronic hemolysis was also anticipated with mitapivat use. At this time, the patient had also initiated post-transplant rejection prophylaxis with mycophenolate, tacrolimus, and prednisone. By December 2022, the patient was still anemic, with a hemoglobin of 7.7 g/dL, and mitapivat was increased to 20 mg twice daily. By January 2023, despite mitapivat therapy, the patient continued to feel fatigued, with a hemoglobin of 7.3 g/dL. At this time, the patient had developed stage 3a chronic kidney disease (CKD) and was started on darbepoetin injections every two weeks to see if this would improve his hemoglobin. In February 2023, with a hemoglobin of 7.7 g/dL and increased fatigue, the patient received one unit of RBC transfusion and mitapivat was increased to the maximum dose of 50 mg twice daily, which he tolerated well.

In March of 2023, in the setting of an upper gastrointestinal bleed confirmed with esophagogastroduodenoscopy, hemoglobin was 6.9 g/dL, and the patient received two units of RBC transfusions. Hemolysis parameters at this time had dramatically improved (bilirubin: 1.8 mg/dL; LDH: 149U/L). In the setting of the patient’s diagnosis of CKD, weekly erythropoietin was initiated (parameter of hemoglobin < 10 g/dL). In April 2023, with a hemoglobin of 7.5 g/dL, the patient reported increasing fatigue and subsequently received one unit of RBC transfusion. In August 2023, after contracting COVID-19, mitapivat was temporarily stopped for five days and hemoglobin dropped to 6.3 g/dL; the patient required urgent RBC transfusion with three units. By September 2023, hemoglobin improved to 11.1 g/dL, direct bilirubin was 2.7 mg/dL, and the patient reported improvement in fatigue. As of January 2024, he has remained transfusion-independent for over six months and continues to tolerate mitapivat.

### 2.3. Case 3

In September 2023, a 29-year-old male sought care for PK-deficiency anemia and transfusional iron overload. At birth, he had neonatal jaundice and underwent a splenectomy when 10 months old and a cholecystectomy later in childhood. The patient had required 3–4 RBC transfusions per year, complicated by secondary hemochromatosis with cirrhosis of the liver for about 4 years prior to arrival at our clinic. He presented for evaluation of liver transplantation at our hospital, which triggered hematology evaluation in our clinic. Prior to that, he was under the care of a pediatric hematologist at an outside facility who treated patients with RBC transfusion as needed, iron chelation therapy, and an eventual trial of mitapivat. The patient was also under pulmonological and cardiological care for portopulmonary hypertension.

At presentation, the patient reported low energy levels. On physical examination, he had scleral icterus. The laboratory parameters are presented in Table 1. The patient had completed a 9-month trial of mitapivat therapy, including a maximum dose of 50 mg twice daily for 3 months, prior to arrival at our clinic. Despite mitapivat therapy, hemoglobin and other hemolytic indices (LDH and reticulocyte counts) and iron indices (ferritin and iron saturation) did not improve, and the patient remained transfusion-dependent. This history of non-response to mitapivat triggered us to order genetic testing of the *PKLR* gene in September 2023, which demonstrated a heterozygous non-missense/non-missense mutation comprising a c.1091dup frameshift mutation on exon 7 and a c.1618+1G>A splice-site mutation on exon IVS10. X-linked hemizygous missense mutation of the *G6PD* gene was also noted. However, the G6PD enzyme level came back normal. It is well described that the non-missense/non-missense genotype responds poorly or does not respond at all to mitapivat therapy. Mitapivat was stopped after nine months due to worsening liver function, worsening cardiopulmonary parameters, and lack of hemoglobin response. The cardiopulmonary and liver dysfunction was attributed to iron overload and not to mitapivat.

## 3. Results and Discussion

PK deficiency is an autosomal recessive inherited condition resulting from mutations in the *PKLR* gene, specific to the liver and RBCs. This mutation results in an impairment of the glycolytic pathway and reduced PK enzyme activity within RBCs. PK deficiency causes a lifelong state of chronic hemolytic anemia. Erythrocytes with defective PK undergo rapid hemolysis in the spleen, giving rise to associated symptoms and complications such as anemia, jaundice, gallstones, thrombosis, and iron overload [1,2]. The clinical diversity of the condition is noticeable in adults, as some patients display minimal anemia symptoms, while others experience more severe symptoms and acquire complications, even in the setting of stable hemoglobin levels, as seen in case 2 [3].

Currently, the management of patients with PK deficiency depends on the age of onset, including phototherapy or exchange transfusion for neonatal hemolysis and hyperbilirubinemia, RBC transfusions, folic acid supplementation, and splenectomy, as well as iron chelation therapy, for both children and adults. Both chronically transfused and transfusion-independent individuals can develop serious complications of chronic hemolysis, including splenomegaly, pigment gallstones, folate deficiency, thrombosis, liver iron overload and cirrhosis, osteoporosis, and extramedullary hematopoiesis [6]. Patients with PK deficiency who require regular RBC transfusions, have severe anemia, or develop massive splenomegaly must undergo splenectomy [7]. Such patients require vaccines, prophylactic antibiotics, and thromboprophylaxis [4]. To optimize the treatment of PK deficiency, it is essential to differentiate complications from the disease itself and those arising as a result of treatments such as splenectomy and RBC transfusions. Iron overload is a significant consequence of PK deficiency, occurring irrespective of transfusion or splenectomy status, but is seen more commonly in patients with a high transfusion burden [2,8]. Furthermore, extramedullary hematopoiesis is a recognized complication of PK deficiency, manifesting as lesions causing spinal cord compression, leg ulcers, and bone marrow abnormalities in older individuals, including myelodysplasia with refractory anemia with ringed sideroblasts and chronic myelomonocytic leukemia [8].

In February 2022, mitapivat (AG-348), an oral allosteric activator of wild-type and mutant RBC PK enzymes, was approved by the FDA as the first treatment for hemolytic anemia in adults with PK deficiency [3]. Specifically, mitapivat works by targeting the underlying enzymatic defect that causes chronic hemolysis and ineffective erythropoiesis by activating and stabilizing mutant PK and increasing PK enzyme activity in patients with PK deficiency [9]. In a randomized phase 2 trial of adults who were not receiving RBC transfusions, 50% of patients treated with mitapivat had an increase of more than 1.0 g/dL in their hemoglobin levels, all of whom had at least one missense mutation [5]. In the patients who demonstrated a hemoglobin response, a decrease in hemolytic markers (absolute reticulocyte count, indirect bilirubin, LDH, and haptoglobin) was also observed [5]. Furthermore, mitapivat is well tolerated. The most common adverse events reported by patients who received therapy for a median of 3 years were headache, insomnia, fatigue, and nasopharyngitis [10]. One important clinical factor to consider is the risk of rebound hemolysis when mitapivat is abruptly discontinued [3]. This has been well described in the literature and was noted in case 2 when mitapivat was suspended for five days due to acute COVID-19 symptoms, triggering an acute rebound hemolysis and severe anemia requiring transfusion.

We have reported two cases of patients with missense/non-missense *PKLR* gene mutations and PK deficiency with varying presentations of hemolytic anemia who responded to treatment with mitapivat and one case of a patient with non-missense/non-missense *PKLR* gene mutation who did not respond to treatment with mitapivat. Case 1 is a young adult who had presented with scleral icterus, low hemoglobin, and elevated hemolysis markers (LDH and total bilirubin). He had no splenectomy or iron overload on presentation. Six years after initial presentation, the patient’s hemoglobin was steadily dropping; the patient had become transfusion-dependent and reported increased fatigue. After initiation of mitapivat, this patient with missense/non-missense *PKLR* gene mutation responded very well, with a significant increase in hemoglobin and a decrease in reticulocyte % (Figure 1). The patient reported improvement in fatigue, and splenectomy was successfully avoided due to the availability of mitapivat. The risk of iron overload for this patient remains minimal due to ongoing mitapivat therapy.

Case 2 is a complex presentation of an older adult who had presented with mild chronic fatigue, scleral icterus, appreciable hepatomegaly, low hemoglobin, and elevated hemolysis markers. The patient required many RBC transfusions, had recurrent episodes of cholangitis, and was iron-overloaded, requiring liver transplantation, complicated by acute kidney injury that progressed to CKD. After initiation of mitapivat, this patient with missense/non-missense *PKLR* gene mutation demonstrated improvement in hemolytic markers, required less frequent RBC transfusions, and reported improvement in fatigue. This patient’s persistent anemia, irrespective of mitapivat therapy, was likely multifactorial, as the patient was taking the immunosuppressive agent mycophenolate in the setting of liver transplantation and had developed CKD. Mycophenolate has been shown to cause anemia by inhibiting erythroid cell line proliferation [11]. In parallel, CKD is a well-studied cause of anemia, with increasing prevalence of anemia among patients with stage 3–5 CKD [12].

Case 3, a young male with non-missense/non-missense *PKLR* gene mutation, PK-deficiency anemia, and transfusional iron overload did not respond to treatment with mitapivat. This patient presented with low hemoglobin and elevated hemolysis markers. He had developed secondary hemochromatosis, cirrhosis of the liver, congestive heart failure with infiltrative cardiomyopathy, and pulmonary arterial hypertension associated with portal hypertension from transfusional iron overload. During the nine-month trial with mitapivat, this patient did not show improvement in hemoglobin or hemolytic markers and continued to require RBC transfusions. The lack of response to therapy with mitapivat in this patient is likely due to the specific genotype present. Mitapivat is well documented to have most favorable responses in individuals carrying missense mutations in the *PKLR* gene, while individuals who are homozygous for *R479H* or possess two non-missense mutations have lower response rates or do not respond at all [5]. This observation further highlights the importance of genetic analysis prior to initiation of therapy with mitapivat, as not all patients will benefit.

### 3.1. Limitations

We present our real-world experience of mitapivat therapy in three adult patients with PK-deficiency hemolytic anemia. The generalization of our findings can be considered a limitation, as variations in patient factors, such as age, sex, other comorbidities, genetic predispositions, and genotype, can lead to different clinical manifestations and responses to treatment. While this case series offers valuable insights into the treatment of PK deficiency with mitapivat for these specific patients with specific genotypes, it is essential to approach the generalization of these findings to a wider population with caution. To improve our comprehension of this treatment and establish guidelines applicable on a broader scale, future research involving larger and more diverse patient cohorts is warranted.

### 3.2. Future Directions

There are investigational therapies being tested to further improve care in hemolytic anemia due to PK deficiency, such as allogeneic hematopoietic stem cell transplant (alloSCT) and gene therapy. While gene therapy (gene editing) remains experimental, alloSCT has been used successfully in clinic and can be considered for patients with severe disease who continue to have a high transfusion burden despite mitapivat therapy and splenectomy.

## 4. Conclusions

Mitapivat stands as a new standard of care for the treatment of symptomatic patients with hereditary hemolytic anemia due to PK deficiency. In patients with PK deficiency, it has demonstrated a favorable safety profile and excellent tolerability. In patients with specific genotypes, mitapivat consistently raises hemoglobin levels; reduces the need for transfusions, thereby potentially reducing iron overload; and improves symptoms such as fatigue. The potential of mitapivat to effectively mitigate, even reverse, the incapacitating complications associated with chronic hemolysis (such as iron overload and cirrhosis of the liver) remains an open question, awaiting further investigation. Thus, there is a pressing need for additional trials incorporating a more comprehensive range of patient factors. These studies will be instrumental in establishing clinical guidelines that can guide best practices and therapeutic approaches to each patient’s unique characteristics.

## Figures and Tables

**Table 1:** Baseline case characteristics.

Characteristics	Case 1	Case 2	Case 3	Normal Range/ Remarks
Age	18	52	28	NA
Gender	M	M	M	NA
Scleral icterus	Present	Present	Present	NA
History of cholelithiasis/gallstones	Yes	Yes	Yes	NA
Cholecystectomy	Yes (age 11)	Yes (age 15)	Yes	NA
Splenectomy	No	Yes (age 2)	Yes (age 10 months)	NA
Iron overload	No	Yes, cirrhosis of liver leading to liver failure and liver transplantation	Yes, cirrhosis of liver, portopulmonary hypertension, awaiting liver transplantation	NA
Iron chelation therapy	No	Yes	Yes	NA
**Laboratory Studies**				
Hb at presentation	9.5	9.1	5.8	13.2–17.1 g/dL
Lowest Hb recorded, triggering mitapivat therapy	6.8	6	5.1	13.2–17.1 g/dL
WBC	4.31	9.7	6.8	3.8–10.8 k/μL
Platelets	207	674	455	140–400 k/μL
LDH	648	229	392	135–225 U/L
Absolute reticulocyte count (reticulocyte %)	226,200 (8.7%)	611,600 (27.8%)	375,900 (36%)	25,000–90,000 cells/μL
Haptoglobin	Not reported	<8	<20	43–212 mg/dL
Bilirubin (total/direct)	10.0/not reported	5.7/0.9	6.7/1.1	0.2–1.2 mg/dL/<0.2 mg/dL
Ferritin	33	2374	1490	30.3–565.7 ng/mL
Transferrin saturation	30	>93	48	20.0–55.0%
Creatinine	0.21	0.70	0.84	0.40–1.30 mg/dL
PK enzyme activity in blood	1.8	2.6	2.2	5.5–12.4 U/g Hb
*PKLR* mutation category (Test performed: hereditary anemia next-generation sequencing (NGS) and deletion/duplication panel)	Missense/non-Missense	Missense/non-Missense	Non-missense/non-missense	**Case 1:** c.721G>T (nonsense)/c.1484C>T (missense) **Case 2:** c.1091dup (frameshift)/c.1493G>A (missense) **Case 3:** c.1091dup (frameshift)/c.1618+1G>A (splice site)
**Clinical Intervention**				
RBC transfusion	Yes	Yes	Yes	NA
Mitapivat therapy	Yes	Yes	Yes	NA
Transfusion status post-mitapivat therapy	Became transfusion-independent immediately	Required less frequent transfusions and eventually became transfusion-independent	Remained transfusion-dependent	NA

Abbreviations: Hb = hemoglobin; LDH = lactate dehydrogenase; M = male; NA = not applicable; PK = pyruvate kinase; RBC = red blood cell; WBC = white blood cell.

## Data Availability

The original contributions presented in the study are included in the article; further inquiries can be directed to the corresponding author.

## References

[1] Bianchi P., Fermo E., Glader B., Kanno H., Agarwal A., Barcellini W., Eber S., Hoyer J.D., Kuter D.J., Maia T.M. (2019). Addressing the diagnostic gaps in pyruvate kinase deficiency: Consensus recommendations on the diagnosis of pyruvate kinase deficiency. Am. J. Hematol..

[2] Bianchi P., Fermo E., Lezon-Geyda K., van Beers E.J., Morton H.D., Barcellini W., Glader B., Chonat S., Ravindranath Y., Newburger P.E. (2020). Genotype-phenotype correlation and molecular heterogeneity in pyruvate kinase deficiency. Am. J. Hematol..

[3] Agios Pharmaceuticals Pyrukynd (mi-Tapivat): Prescribing Information 2022. https://www.accessdata.fda.gov/drugsatfda_docs/label/2022/216196s000lbl.pdf.

[4] Al-Samkari H., Galactéros F., Glenthøj A., Rothman J.A., Andres O., Grace R.F., Morado-Arias M., Layton D.M., Onodera K., Verhovsek M. (2022). Mitapivat versus Placebo for Pyruvate Kinase Deficiency. N. Engl. J. Med..

[5] Grace R.F., Rose C., Layton D.M., Galactéros F., Barcellini W., Morton D.H., van Beers E.J., Yaish H., Ravindranath Y., Kuo K.H. (2019). Safety and Efficacy of Mitapivat in Pyruvate Kinase Deficiency. N. Engl. J. Med..

[6] Grace R.F., Zanella A., Neufeld E.J., Morton D.H., Eber S., Yaish H., Glader B. (2015). Erythrocyte pyruvate kinase deficiency: 2015 status report. Am. J. Hematol..

[7] Grace R.F., Mark Layton D., Barcellini W. (2019). How we manage patients with pyruvate kinase deficiency. Br. J. Haematol..

[8] Boscoe A.N., Yan Y., Hedgeman E., van Beers E.J., Al-Samkari H., Barcellini W., Eber S.W., Glader B., Yaish H.M., Chonat S. (2021). Comorbidities and complications in adults with pyruvate kinase deficiency. Eur. J. Haematol..

[9] Kung C., Hixon J., Kosinski P.A., Cianchetta G., Histen G., Chen Y., Hill C., Gross S., Si Y., Johnson K. (2017). AG-348 enhances pyruvate kinase activity in red blood cells from patients with pyruvate kinase deficiency. Blood.

[10] Grace R.F., Layton D.M., Galactéros F., Barcellini W., van Beers E.J., Yaish H.M., Ravindranath Y., Kuo K.H.M., Sheth S., Kwiatkowski J.L. (2019). Long-term safety and efficacy of mitapivat (AG-348), a pyruvate kinase activator, in patients with pyruvate kinase deficiency: The DRIVE PK Study. Blood.

[11] Pile T., Kieswich J., Harwood S., Yaqoob M.M. (2011). A possible explanation for anemia in patients treated with mycophenolic acid. Transplantation.

[12] St Peter W.L., Guo H., Kabadi S., Gilbertson D.T., Peng Y., Pendergraft T., Li S. (2018). Prevalence, treatment patterns, and healthcare resource utilization in Medicare and commercially insured non-dialysis-dependent chronic kidney disease patients with and without anemia in the United States. BMC Nephrol..

